# Impact of milk secretor status on the fecal metabolome and microbiota of breastfed infants

**DOI:** 10.1080/19490976.2023.2257273

**Published:** 2023-09-23

**Authors:** Aidong Wang, Aly Diana, Sofa Rahmannia, Rosalind S Gibson, Lisa A Houghton, Carolyn M Slupsky

**Affiliations:** a Department of Food Science and Technology, University of California, Davis, CA, USA; b Department of Public Health, Faculty of Medicine, Universitas Padjadjaran, Bandung, Indonesia; c Nutrition Working Group, Faculty of Medicine, Universitas Padjadjaran, Bandung, Indonesia; d Faculty of Medicine, Universitas Pasundan, Bandung, Indonesia; e School of Population and Global Health, University of Western Australia, Crawley, Western Australia, Australia; f Department of Human Nutrition, University of Otago, Dunedin, New Zealand; g Department of Nutrition, University of California, Davis, CA, USA

**Keywords:** Breast milk, secretor status, oligosaccharides, exclusive breastfeeding, infant, microbial metabolism, microbiota

## Abstract

Maternal secretor status has been shown to be associated with the presence of specific fucosylated human milk oligosaccharides (HMOs), and the impact of maternal secretor status on infant gut microbiota measured through 16s sequencing has previously been reported. None of those studies have confirmed exclusive breastfeeding nor investigated the impact of maternal secretor status on gut microbial fermentation products. The present study focused on exclusively breastfed (EBF) Indonesian infants, with exclusive breastfeeding validated through the stable isotope deuterium oxide dose-to-mother (DTM) technique, and the impact of maternal secretor status on the infant fecal microbiome and metabolome. Maternal secretor status did not alter the within-community (alpha) diversity, between-community (beta) diversity, or the relative abundance of bacterial taxa at the genus level. However, infants fed milk from secretor (Se+) mothers exhibited a lower level of fecal succinate, amino acids and their derivatives, and a higher level of 1,2-propanediol when compared to infants fed milk from non-secretor (Se-) mothers. Interestingly, for infants consuming milk from Se+ mothers, there was a correlation between the relative abundance of *Bifidobacterium* and *Streptococcus*, and between each of these genera and fecal metabolites that was not observed in infants receiving milk from Se- mothers. Our findings indicate that the secretor status of the mother impacts the gut microbiome of the exclusively breastfed infant.

## Introduction

Although the total concentration of oligosaccharides in human milk has low biological variability, on any given day the concentrations of individual human milk oligosaccharides (HMOs) exhibit high inter-individual variation.^1,2^ Variation in the concentration of individual oligosaccharides is driven by maternal genetics, stage of lactation, as well as other unknown factors.^3,4^ The secretor (Se) gene, *fut2*, codes for α-1,2-fucosyltransferase 2 (FUT2), which is responsible for producing HMOs such as 2’-fucosyllactose (2’FL), lacto-N-fucopentaose I (LNFP I), and lactodifucotetraose (LDFT).^5^ For women with a functional FUT2 enzyme (Se+), 2’FL is the most abundant HMO in their breast milk, whereas for women with a nonfunctional FUT2 enzyme (Se-), 2’FL has been shown to be below detection limits.^1,6^ Some studies have reported that for mothers with Se- status, α-1,2-fucosyltransferase 1 (FUT1) can synthesize 2’FL, albeit at low levels.^4,7^ Nonetheless, breastmilk from Se- mothers has been reported to have lower concentrations of fucosylated HMOs, total HMOs, and higher levels of non-fucosylated neutral HMOs when compared to breastmilk from Se+ women.^1,6,8^

The difference in HMO profiles between women with a Se+ or Se- phenotype poses the question as to whether there may be selective advantages for the infant. As HMOs are important substrates for microbial fermentation, the impact of secretor status on infant gut microbial composition has been the focus of many studies. One such study reported that in premature infants fed Se+ milk, a trend toward lower levels of *Proteobacteria* and higher levels of *Firmicutes* was observed in the fecal microbiome compared to premature infants fed Se- milk.^9^ Two separate studies showed that Se+ milk consumption was associated with a higher abundance and faster colonization of *Bifidobacterium* in the gut of term breastfed infants.^10,11^ The impact of maternal secretor status on gut microbial composition has been reported to persist up to the age of 2–3 years, where children of secretor mothers have higher *Bifidobacterium* and lower *Bacteroides* than children of non-secretor mothers.^12^ However, these associations have not been observed in all studies. One study reported no impact of maternal secretor status on infant gut microbiota when partially or exclusively breastfed (EBF) infants were born vaginally, but a shift in microbial composition was observed in cesarean-born infants.^13^ Another study reported no impact of maternal secretor status on overall breastfed infant gut microbial community or *Bifidobacterium* amplicon sequence variants (ASVs).^14^ A previous study reported a negative correlation between milk 2’FL and gut *Bifidobacterium* in EBF infants.^15^

Although studies have investigated the impact of maternal secretor status on infant microbial composition, few have reported whether it alters the gut microbial fermentation capability. It is of great interest to understand if this difference in the milk metabolome based on maternal secretor status has an impact on the production of microbial fermentation products. While previous reports investigating the impact of maternal secretor status on the infant gut microbiome have taken into account infant feeding practices, the determination of EBF status was solely based on mother’s self-reporting and was not confirmed through objective techniques, such as the dose-to-mother (DTM) deuterium-oxide method.^8–14^ A previous study investigating the EBF rate of Guatemalan mothers showed that while the self-reported EBF rates were 50% (report of current feeding practice) and 61% (by 24-h recall), the EBF rate measured via the DTM method was only 36%.^16^ A separate study reported that based on the DTM technique, 75% of the self-reported EBF infants were fed food other than breastmilk.^17^ It is important to confirm EBF status with an objective technique such as the DTM method to reduce the confounding impact of food and water intake on the infant gut microbiome. In the present study, we evaluated the impact of secretor status on infant gut microbial composition and the fecal metabolome with EBF status confirmed through the DTM method.

## Results

The present study aimed to compare the gut microbiota and fecal metabolome between EBF infants receiving milk from phenotypically Se+ and Se- mothers. Only samples from those mother-infant pairs where EBF was confirmed through the DTM method, and from which infant fecal and maternal breastmilk samples were collected, were used in this study. After data generation, a few fecal samples were excluded because of suspected sequencing errors (*n* = 2) or suspected urine contamination (*n* = 5, determined by the presence of urea in the NMR spectrum). In the end, data from a total of 160 mother-infant pairs were included in the analysis.

**Figure 1.**
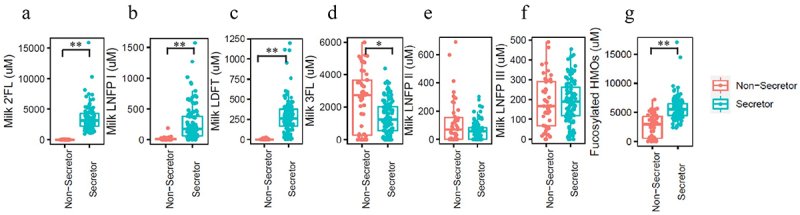
Representative fucosylated milk oligosaccharides measured in breast milk from mothers in this study. (a) 2’-fucosyllactose (2’FL), (b) lacto-N-fucopentaose I (LNFP I), (c) lactodifucotetraose (LDFT), (d) 3-fucosyllactose (3FL), (e) lacto-N-fucopentaose II (LNFP II), (f) lacto-N-fucopentaose III (LNFP III), (g) the summation of (a)-(f).

Although the maternal secretor genotype was not determined, milk secretor phenotype was determined through analysis of quantified oligosaccharides (2’FL, 3-fucosyllactose (3FL), lacto-N-fucopentaose I (LNFP I), lacto-N-fucopentaose II (LNFP II), lacto-N-fucopentaose III (LNFP III), and lactodifucotetraose (LDFT), Supplementary Figure S1, Supplementary Table S1), and defined as Se+: [2’FL] ≥200 μM; and Se-: [2’FL] <200 μM (Figure 1a). Lewis blood type associated HMOs (3FL, LNFP II and LNFP III) were also measured (Figure 1d–f). As described previously,^18^ based on these data, two of the mothers were classified as phenotypically Lewis negative (one in the Se+ group, and one in the Se- group).

As shown in Table 1, 113 of the mother-infant pairs were assigned to the Se+ group, while 47 were assigned to the Se- group. Maternal age and body mass index (BMI, kg/m^2^), infant age, length-for-age, weight-for-age, weight-for-length, and infant BMI-for-age *z*-scores were comparable between the two groups. Morbidity data reflected the prevalence of disease symptoms over the last 7 days prior to sample collection. A total of 14.2% of infants in the Se+ group reported cough, while the prevalence of these symptoms in the Se- group was significantly higher at 34.0% (*p* = .004, chi-square test). The prevalence of vomiting, fever, diarrhea, and tachypnea in the last 7 days was similar between the two groups.

**Table 1.** Characteristics of mother and infant subjects based on mother’s milk secretor status.

Characteristics	Milk secretor status phenotype		
	Secretor	Non-secretor	*p*-value
Sample size, *n*	113	47	
**Mother**			
Age, year	26.2 (6.0)	25.2 (5.8)	.279
BMI, kg/m^2^	24.5 (3.6)	24.0 (3.3)	.486
**Infant**			
Sex, %female	60.20%	46.80%	.640
Age, months	3.5 (1.1)	3.5 (1.1)	.805
Length, cm	58.8 (6.0)	59.4 (3.1)	.874
Weight, kg	6.1 (0.9)	6.4 (1.0)	.356
LAZ	−1.00 (0.87)	−1.05 (0.82)	.474
WAZ	−0.36 (0.89)	−0.30 (0.89)	.630
WLZ	0.65 (1.02)	0.75 (1.06)	.395
BMIZ	0.29 (0.97)	0.41 (1.04)	.240
Vomit, *n* (%) #	8 (7.1%)	3 (6.4%)	.874
Fever, *n* (%) #	19 (16.8%)	8 (17.0%)	.975
Diarrhea, *n* (%) #	9 (8.0%)	5 (17.0%)	.586
Cough, *n* (%) #	16 (14.2%)	16 (34.0%)	.004
Tachypnea, *n* (%) #	4 (3.5%)	2 (4.3%)	.828

Mother’s age, body mass index (BMI), infant age, length, weight, length-for-age *z*-score (LAZ), weight-for-age *z*-score (WAZ), weight-for-length *z*-score (WLZ), and infant BMI-for-age *z*-score (BMIZ) are shown as mean (standard deviation). *P*-values were calculated using the Mann–Whitney U test.

#Morbidity data reflect the prevalence of symptoms over a 7-day period prior to sample collection. *P*-values for morbidity data and infant sex were calculated using the chi-square test.

**Figure 2.**
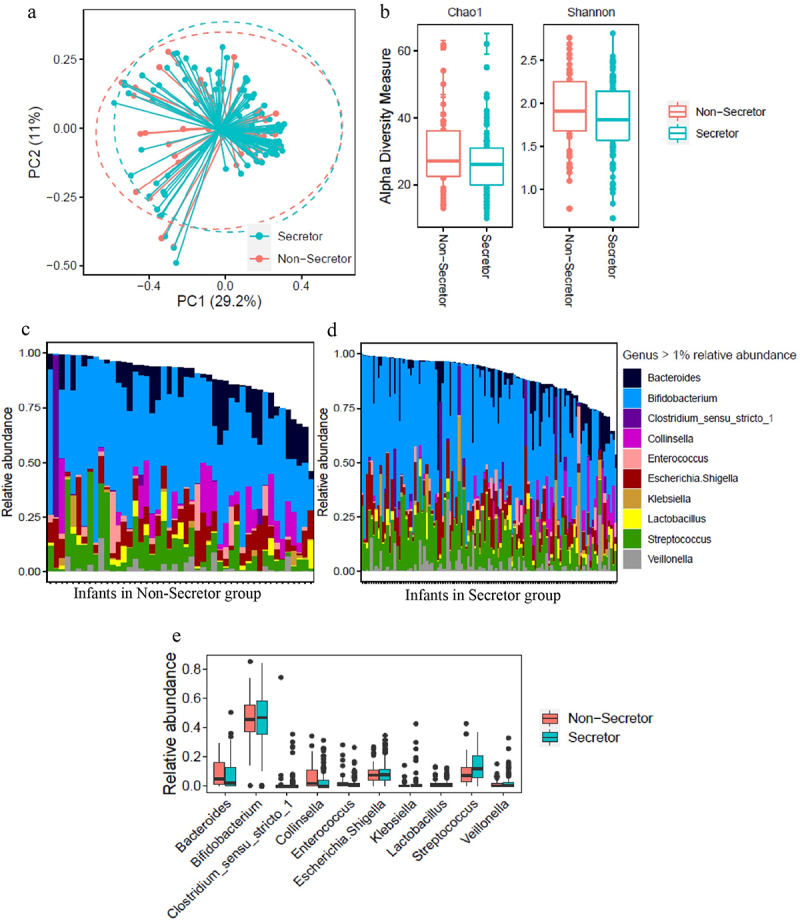
Comparison of fecal microbiome data in infants consuming secretor (cyan) or non-secretor (red) milk. (a) Analysis of beta-diversity using Principal coordinates analysis (PCoA) based on bray distance matrix data. The centroids of each cluster (centroid of mass) were calculated as the average PC1 and PC2 of all samples for each group. The ellipses were constructed based on multivariate normal distribution at 95% confidence level. (b) Analysis of alpha diversity represented as Chao1 and Shannon index. (c) The relative abundance of genera represented at a level of over 1% in the feces of infants consuming secretor milk. (d) The relative abundance of genera represented at a level of over 1% in the feces of infants consuming non-secretor milk.

To explore the impact of maternal secretor phenotype on infant fecal microbiota, 16S rRNA sequencing was performed. PCoA showed no clear separation in the microbiome between infants receiving Se+ and Se- milk (Figure 2a). Chao1 and Shannon alpha diversity showed no significant differences between the Se+ and Se- groups via the Mann–Whitney test (Chao1 *p* = 0.12 and Shannon *p* = 0.21, Figure 2b). Figure 2c, d shows those genera with over 1% relative abundance for each of the infants from both groups, and Figure 2e shows a comparison of the average relative abundance of each genus. As expected, *Bifidobacterium* was the major genus, and the abundance was similar between infants consuming milk from Se+ and Se- mothers. Other genera present in significant amounts included *Bacteroides*, *Collinsella*, *Streptococcus*, and *Veillonella*, and no difference between the relative abundance of these taxa or other taxa with over 1% relative abundance was observed in the feces of infants consuming milk from Se+ or Se- mothers. Since infant age was between 2.5 and 5.5 months, we re-analyzed the relative abundance data by dividing the infants into two age groups (younger or older than 3.75 months) ensuring an equal number of subjects between Se+ and Se- groups to see if age played a role (Supplementary Figure S2). While we observed more taxa at a relative abundance over 1% in infants at 3.75 months and older, no difference in genera were observed between infants consuming Se+ or Se- milk.

**Figure 3.**
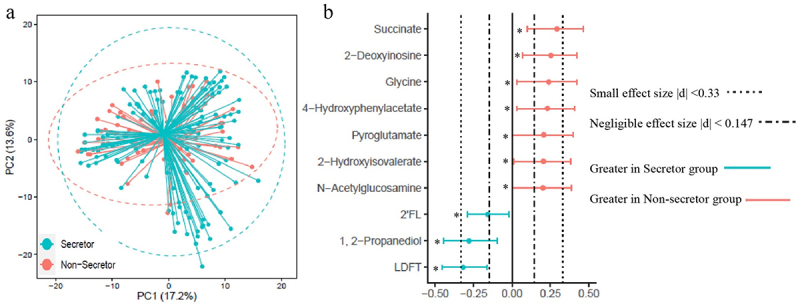
Comparison of infant fecal metabolome data in infants consuming secretor (cyan) or non-secretor (red) milk. (a) Principal coordinate analysis (PCoA) and (b) forest plot illustrating the Cliff’s delta effect size analysis of individual metabolites. Metabolites with a *p* < .05 determined using the Mann–Whitney U-test with FDR correction are indicated with an asterisk.

Quantified fecal metabolites (Supplementary Table S2) were examined to evaluate the impact of maternal secretor phenotypes on intestinal microbial fermentation products. PCoA of fecal metabolites showed no clear separation between infants of moms in the Se+ and Se- groups (Figure 3a). To determine if subtle differences in specific metabolites could be detected, fecal metabolites were compared using a combination of effect size (determined using Cliff’s δ) and the Mann-Whitney U-test with FDR correction (Figure 3b). 2’FL, LDFT, and 1,2-propanediol were significantly higher in infant feces from the Se+ group compared to the Se- group (*p* < .05, with a small effect size (0.16 < |δ| <0.32)). Another HMO-related structure, N-acetylglucosamine (GlcNAc) was significantly lower in the Se+ group (*p* = .05, δ = 0.20). Amino acids and related metabolites (glycine, pyroglutamate, 4-hydroxyphenylacetate, and 2-hydroxyisovalerate) were also significantly lower in the Se+ group (*p* < .05, 0.20 < δ < 0.24 for all), as was succinate and 2’-deoxyinosine (*p* < .05, 0.25 < δ < 0.29). To see if infant age (between 2.5 and 5.5 months) impacted the fecal metabolome between the Se+ and Se- groups, we divided the infants into two age groups (younger or older than 3.75 months) ensuring an equal number of subjects in the Se+ and Se- groups, and approximately equal representation of males and females. No metabolites were significantly different between infants in the Se+ and Se- groups at either age assessed by the Mann-Whitney U-test with FDR correction, suggesting that a sample size <30 is not powered enough to observe differences in the fecal metabolome between infants receiving Se+ milk and those receiving Se- milk. A forest plot showing Cliff’s δ effect size (Supplementary Figure S3) comparing Se+ and Se- infants less than 3.75 months of age and greater than 3.75 months of age revealed similar differences as when comparing infants in the Se+ and Se- groups in the entire cohort (higher succinate, 2’-deoxyinosine, 4-hydroxyphenylacetate, 2-hydroxyisovalerate, glycine and pyroglutamate in infants in the Se- group and higher 2’-FL, 1,2-propanediol and LDFT in the Se+ group).

**Figure 4.**
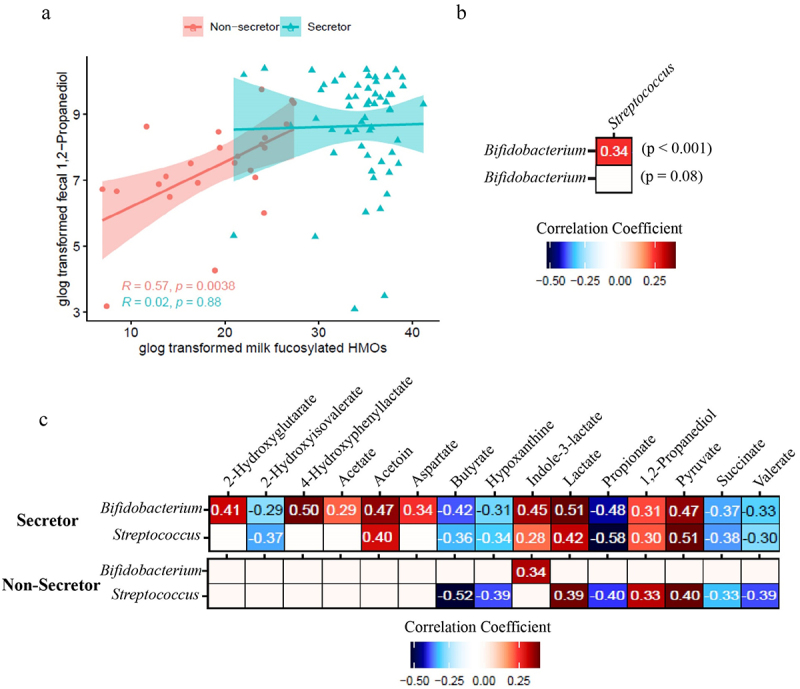
Correlation between the fecal microbiome and fecal metabolome of infants receiving secretor or non-secretor milk. (a) Association between total milk fucosylated HMO concentration in milk from secretor (cyan) and non-secretor (red) mothers and infant fecal 1, 2-propanediol concentrations. (b) Spearman correlation between bacterial taxa in the feces of infants consuming milk from secretor and non-secretor mothers. (c) Spearman correlation between fecal metabolites and the relative abundance of *Bifidobacterium* and *Streptococcus* of infants consuming milk from secretor and non-secretor mothers.

To further investigate the association between milk HMO, fecal microbiota, and the metabolome, Spearman correlations were conducted between fecal metabolites and milk HMOs, and between fecal metabolites and microbiome relative abundance data. Fecal 1,2-propanediol concentration, a fermentation byproduct shown to be produced by multiple *Bifidobacterium* species,^19^ was positively correlated with the concentration of total milk fucosylated HMOs in the Se- group (*p* < .05) but not in the Se+ group (Figure 4a). Interestingly, no correlation between milk fucosylated HMOs and *Bifidobacterium* relative abundance was observed (data not shown). Since there was an unbalanced sample size, we re-analyzed the samples utilizing a similar number of subjects in the Se+ and Se- groups and a similar result was obtained (Supplementary Figure S4).

A positive correlation was observed between the relative abundance of *Bifidobacterium* and *Streptococcus* (*p* < .001) in infants in the Se+ group but not in the Se- group (Figure 4b). Interestingly, *Bifidobacterium* and *Streptococcus* had similar correlations with fecal metabolites in the Se+ group but not in the Se- group (Figure 4c). In the Se+ group, the relative abundance of *Bifidobacterium* and *Streptococcus* was negatively correlated with several short chain fatty acids and organic acids including butyrate (*p* < .001), propionate (*p* < .001), valerate (*p* < .001), and succinate (*p* < .001), as well as the amino acid breakdown metabolite 2-hydroxyisovalerate (*p* < .01 for *Bifidobacterium* and *p* < .001 for *Streptococcus*) and a purine degradation metabolite, hypoxanthine (*p* < .01 for *Bifidobacterium* and *p* < .001 for *Streptococcus*). *Bifidobacterium* and *Streptococcus* relative abundances were both positively correlated with acetoin (*p* < .001), indole-3-lactate (*p* < .001 for *Bifidobacterium* and *p* < .05 for *Streptococcus*), lactate (*p* < .001), 1,2-propanediol (*p* < .001 for *Bifidobacterium* and *p* < .01 for *Streptococcus*) and pyruvate (*p* < .001) in the Se+ group. *Bifidobacterium* relative abundance was also positively correlated with 4-hydroxyphenyllactate (*p* < 0.001), 2-hydroxyglutarate (*p* < .001), acetate (*p* < .01), and aspartate (*p* < .001) in the Se+ group. In the Se- group, *Bifidobacterium* relative abundance was positively correlated with indole-3-lactate (*p* < 0.05), whereas *Streptococcus* relative abundance was positively correlated with lactate (*p* < .01), 1,2-propanediol (*p* < .05), pyruvate (*p* < .01), and negatively correlated with butyrate (*p* < .001), hypoxanthine (*p* < .01) propionate (*p* < .01), succinate (*p* < .05), and valerate (*p* < .01).

## Discussion

HMOs have been reported to have beneficial effects on infants through shaping infant gut microbial composition. Receiving Se+ milk has been reported to be positively associated with gut *Bifidobacterium* in some the US,^10^ Australian,^12^ and Chinese infants,^11^ not correlated with gut microbiota in some Finnish vaginally born,^13^ and Danish infants,^14^ and negatively associated with *Bifidobacterium* in some EBF US infants.^15^ One thing in common with all these studies is that reports of exclusive feeding were solely based on maternal self-report. Moreover, these studies mostly took place in resource-rich countries. Our study on EBF infants was performed in a rural setting in Indonesia, and exclusive breastfeeding was validated through the deuterium oxide DTM method. Here, no significant difference in gut microbiota between infants receiving Se+ and Se- milk based on results from PCoA (Figure 2a), alpha diversity measurement (Figure 2b), and comparison of microbial genera (Figure 2e) was observed.

We speculate that geographic and/or socioeconomic factors may play a role in how maternal secretor status shapes infant gut microbial composition and accommodation to the available HMOs entering the gut. Indeed, a difference in infant gut microbiome between WEIRD (western, educated, industrialized, rich, and democratic) societies and other parts of the world has recently drawn attention. Specifically, a loss of highly specialized *Bifidobacterium* species in the infant gut and an increase in fecal pH have been recognized in resource-rich countries over the past century.^20^ *Bifidobacterium longum* subsp. *infantis*, which has a complete capacity for HMO utilization, was found to be rare in US infants.^21^ Though without direct evidence, interventions such as provision of antibiotics and cesarean section have been speculated to be part of the reason causing the loss of this bacterial taxon.^22,23^ All infants in the present study were from a rural area in Indonesia, and the sample size reported here is much larger (160 infants) compared to previously published studies (22 to 76 infants).^9–14^

Although no difference in the genus level from the 16s rRNA gene sequencing results was observed, there could be a shift of *Bifidobacterium* at the species level due to the difference in HMO profile (Figure 1a–g). Different species of *Bifidobacterium* have been reported to have different capability and preference for utilizing HMOs.^24^ For instance, several *Bifidobacterium* subspecies (including *Bifidobacterium longum* subsp. *infantis*, *Bifidobacterium longum* subsp. *suis* BSM11–5, and *Bifidobacterium kashiwanohense*) have been reported to grow in the presence of 2’FL and 3FL,^19^ whereas *Bifidobacterium breve* KA179 was reported to grow on 2’FL but not on 3FL.^25^ The difference in concentration of 1,2-propanediol in infant feces (Figure 3b) between infants consuming milk from Se+ or Se- mothers suggests that there are differences at the species and strain levels of *Bifidobacterium*. 1,2-Propanediol has been reported to be produced by *Bifidobacterium longum* subsp. *infantis* and *Bifidobacterium longum* subsp. *suis* BSM11–5 but not by *Bifidobacterium kashiwanohense* DSM 21854 when grown on 2’FL and 3FL.^19,26,27^ The higher level of 1,2-propanediol in the feces of infants fed Se+ milk is likely a result of colonization of *Bifidobacterium* species that can produce 1,2-propanediol from 2’FL. Importantly, *Bifidobacterium* was positively correlated with 1,2-propanediol in infants fed milk from Se+ mothers but not Se- mothers (Figure 4c).

It is worth noting that 1,2-propanediol was still present in the feces of infants fed milk from Se- mothers. This could be attributed to species such as *B. longum* subsp. *suis* BSM11–5 which synthesize 1,2-propanediol mainly from 3FL when given both 2’FL and 3FL.^19^ Furthermore, 1,2-propanediol showed a positive correlation with fucosylated HMOs in the Se- group but not in the Se+ group (Figure 4a). This suggests that the production of 1,2-propanediol arises from the fermentation of fucosylated HMOs and that the production may be slower when 2’FL is not present. Once a certain amount of 1,2-propanediol is produced, it could be that a “steady-state” is reached. Whether this “steady state” is due to increased absorption or utilization by other microbes remains to be studied.^28^

A lower level of succinate as well as amino acids and related metabolites (glycine, pyroglutamate, 4-hydroxyphenylacetate, and 2-hydroxyisovalerate) (Figure 3b) in the feces of infants fed Se+ milk may suggest a shift in carbohydrate and amino acid fermentation. There were no significant differences in fecal organic acids associated with colonic pH (other than succinate), so it is unlikely that there are differences in stool pH between the groups (although this was not specifically measured). Lower overall levels of fucosylated HMOs in the milk could lead to slight changes in the fermentative capacity of the microbiome, and thus slight changes in the abundance of certain bacterial taxa resulting in the differences observed here. Higher fecal succinate has been associated with inflammatory bowel disease (IBD),^29^ and succinate accumulation has been attributed to changes in the abundance of succinate-consuming gut microbes.^30^ One study suggested that higher succinate in the neonate may favor the colonization of strict anaerobes.^31^ The potential impact of succinate in the neonatal gut on infant health needs further study.

Breastfeeding has been reported to decrease respiratory infection in both infancy and childhood,^32,33^ with the benefit attributed to the antiadhesive function of HMOs.^34^ 2’FL and lacto-N-neotetraose (LNnT) supplemented infant formula was shown to decrease the risk of lower respiratory tract infection in infants,^35^ and 2’FL was shown to be the most effective HMO in decreasing respiratory syncytial virus load and cytokines in epithelial cells when compared to 3FL, 6’-sialyllactose (6’SL) and other oligosaccharides.^36^ In this study, cough was more common in infants fed Se- milk (34.0%) compared to infants fed Se+ milk (14.2%) (*p* = .004, chi-square test) (Table 1).

A positive association between *Bifidobacterium* and *Streptococcus* (Figure 4b) and a similar trend of correlation with fecal metabolites between the two microbes (Figure 4c) in the Se+ group indicate that there could be crosstalk between these two microbial taxa in infants fed Se+ milk. A previous study reported a synergistic effect between *Bifidobacterium lactis* and *Streptococcus thermophilus* when cultured in skim milk, and such positive mutual interaction was further improved by inulin due to its prebiotic and bifidogenic function.^37^ Specifically, better growth of both organisms was observed in co-culture compared to pure cultures, and the increase in the biomass of the two microbes was larger in the presence of inulin.^37^ In the present study, Se+ associated HMOs may act as prebiotics favoring the growth of certain species of *Bifidobacterium* and *Streptococcus* that have a synergistic relationship. Indeed, we previously showed that *Streptococcus* is one of the core fecal taxa in infants during the exclusive feeding period between 2 and 6 months of age.^38^

The positive correlation between *Bifidobacterium* and 4-hydroxyphenyllactate in the Se+ group but not in the Se- group further delineates differences at the species and strain level of *Bifidobacterium* between the two groups. 4-Hydroxyphenyllactate, a tyrosine metabolite, has been shown to be produced by *Bifidobacterium in vitro*.^39,40^ A recent study showed that 4-hydroxyphenyllactate was primarily produced by *Bifidobacterium* species that utilize HMOs including *Bifidobacterium bifidum*, *Bifidobacterium breve*, *Bifidobacterium longum* subsp. *longum*, *Bifidobacterium longum* subsp. *infantis* and *Bifidobacterium sardovii* via aromatic lactate dehydrogenase.^41^ Other *Bifidobacterium* species such as *Bifidobacterium adolescentis* and *Bifidobacterium pseudocatenulatum* have not been observed to produce 4-hydroxyphenyllactate.^41^ Interestingly, the fecal concentration of 4-hydroxyphenyllactate was not significantly different between infants from Se+ and Se- mothers, which suggests that other bacterial species may be creating this compound. Indeed, 4-hydroxyphenyllactate has been shown to be produced by other microbes including *Lactobacillus fermentum* and *Eubacterium lentum*.^39^

*Bifidobacteria* have been reported to be capable of producing lactate,^37,42^ and their ability to produce lactate could be affected by the availability of different HMOs. Specifically, *Bifidobacterium longum* JCM 1260, JCM 7011, and JCM 7009 produce lactate in the presence of 2’FL but not 3FL.^43^ This could shed light on the presence of the positive correlation between *Bifidobacterium* and lactate in the Se+ group but not in the Se- group. The utilization of lactate by microbes such as *Veillonella* in the infant gut could also be a contributing factor. *Streptococcus thermophilus* ATCC19258, a common human gut microbe, was reported to utilize either 2’FL or 3FL to produce lactate.^43^ This could explain the positive association between *Streptococcus* and lactate in both the Se+ and Se- groups.

The tryptophan metabolite indole-3-lactate has been shown to be produced by *Bifidobacterium longum subsp. infantis* in the presence HMOs.^44^ However, the production of indole-3-lactate is not necessarily dependent on the presence of HMOs, as multiple *Bifidobacterium* species (including *Bifidobacterium longum* subsp. *longum*, *Bifidobacterium longum* subsp. *infantis*, *Bifidobacterium breve*, and *Bifidobacterium bifidum*) are able to produce indole-3-lactate when cultured in DeMan, Rogosa and Sharpe (MRS) medium.^45^ This could explain the presence of the positive association between *Bifidobacterium* in general (Figure 4c) and indole-3-lactate in both Se+ and Se- groups, and a similar concentration of fecal indole-3-lactate between the two groups. Indole-3-lactate has been reported to be anti-inflammatory,^44^ and has been shown to be negatively correlated with calprotectin in premature infant feces.^46^

The results obtained in the present study show the impact of maternal secretor status on infant gut microbial composition and their fermentation capability. Distinct HMO profiles between breast milk from Se+ and Se- women were confirmed. Though previous studies have reported differences in infant microbial composition when consuming Se+ milk and Se- milk,^8–11^ no significant difference in the infant gut microbiome based on 16s sequencing was observed in this study, indicating that there could be differences at the species and strain level, and further that there may be other factors playing a role in shaping the gut microbiome and its function.

One limitation of the current study is that the secretor status of the infants was not measured. Studies have reported that being a Se- infant was associated with resistance against diarrhea prevalence and norovirus.^47,48^ Therefore, information on the secretor status of both mothers and the infants could provide a better understanding of how HMOs function to protect infants. Future studies should also investigate the microbiome with higher resolution to evaluate specific species and strains of *Bifidobacterium* and study the interaction of *Bifidobacterium* and *Streptococcus* at the species or strain level.

## Materials and methods

The current study included a total of 160 mother–infant pairs with infant postnatal age from 2 to 5.5 months from the Sumedang district in the province of West Java, Indonesia. The inclusion criteria for infants included gestational age and health status and was described in a previous study.^49^ The anthropometric and morbidity status of the mothers and their infants were measured as described previously.^50^ Ethical approval for the study was granted by the University of Otago Human Research Ethics Committee New Zealand (H15/125) and the Health Research Ethics Committee Faculty of Medicine Universitas Padjadjaran, Bandung (081/UN6.C1.3.2/KEPK/PN/2016), Indonesia.

### Breastfeeding status determination

The determination of the EBF status was based on the dose-to-mother (DTM) method described previously.^49,50^ Briefly, mothers were provided a dose of deuterium oxide, and saliva samples were collected from mother-infant pairs over the course of 14 days. Using these data, infant daily water intake from sources other than breastmilk was calculated and then compared to a cut-off value of 86.6 g/d to classify EBF status over the 14-day collection period.^49^

### Breast milk sample collection and metabolite extraction

Full expression of morning milk samples was collected using breast pumps (Harmony, Medela, Baar, Switzerland) after instructing the mothers to take strict precautions to avoid all sources of contamination. After gentle mixing and aliquoting, milk samples were stored at −80°C until analysis.

For metabolomics analysis, milk samples were thawed on ice, vortexed, and centrifuged at 12k rcf, at 4°C for 5 min to separate milk lipids. A total of 350 μL of the aqueous phase was transferred to a pre-washed 3 kDa Amicon filter (Amicon ultra centrifugal filter, Millipore, Billerica, MA) to remove lipids and proteins. To 207 μL of filtrate, 23 μL of internal standard (5 mM 3-(trimethylsilyl)-1-propanesulfonic acid-d6 (DSS-d6) in 99.8% D_2_O (to serve as a lock) and 0.2% NaN_3_ (to inhibit bacterial growth)) was added. To minimize pH-based peak movement in the NMR spectra, the pH of each sample was adjusted to 6.85 ± 0.07 by adding small amounts of NaOH or HCl. A total of 180 μL of the mixture was transferred to 3 mm Bruker NMR tubes (Bruker, Billerica, MA) and stored at 4°C until spectral acquisition.

### Fecal sample collection and metabolite extraction

Fresh fecal samples from infants were collected into Eppendorf tubes directly from the nappy and stored at −80°C until analysis. To prepare for microbiome and metabolome analysis, fecal samples were thawed on ice, and fecal metabolites were extracted as previously described.^38^ Briefly, 250 mg of fecal material was combined with 1.5 mL of ice-cold Dulbecco’s phosphate buffered saline (DPBS, 1X, pH 7.4) for metabolite extraction. After vortexing and centrifugation (14 k crf, 4°C, for 5 min), the supernatant was filtered through a syringe filter (0.22 µm pore size, Millex-GP syringe filter, Millipore, Billerica, MA) followed by an Amicon filter (3 kDa). Samples were prepared as above for NMR analysis. The pellet was collected and saved at 4°C for DNA extraction (described below).

To estimate the water content of each sample, approximately 75 mg of feces was weighed into a 2 mL screw-cap tube and lyophilized (Labconco FreeZone 4.5 L Freeze Dry System, Labconco, Kansas City, MO). The weight of the tube was analytically determined before and after drying and used below for calculating the amount of water in the extracted sample.

### NMR acquisition, data processing, and quantification

^1^H NMR spectra were acquired at 298 K using a NOESY ^1^H presaturation experiment (‘noesypr1d’) on a Bruker Avance 600 MHz NMR spectrometer (Bruker BioSpin, Germany) equipped with a SampleJet autosampler (Bruker BioSpin, Germany) as previously described.^38^ After manual phasing and baseline correction in Chenomx processor, a total of 97 fecal metabolites (Supplementary Table S2) were quantified using Chenomx Profiler (Chenomx NMR Suite v8.3, Chenomx Inc, Edmonton, Alberta, Canada) based on the established method of targeted profiling.^51^ The resulting metabolite concentrations were corrected based water content as described in our previous study,^38^ where fecal water estimate was calculated for each sample as follows:

(1) $$\begin{array}{rl} & Fecal\;water\;estimate=\left(1-\frac{Dry\;weight}{Wet\;weight}\right)\\ & \;\;\;\;\;\;\;\;\;\;\;\;\;\;\;\;\;\;\;\;\;\;\;\;\;\;\;\;\;\;\;\;\;\;\;\;\;\;\;\;\times Weight\;of\;extracted\;sample \end{array}$$

### Milk secretor status phenotype determination and oligosaccharide quantification

Milk secretor status determination was based on the presence or near absence of 2’FL in the NMR spectra of human milk (Supplementary Table S1), which was identified and quantified from an NMR spectral library created through the analytical preparation of commercially available HMO standards as previously described.^1,18^ Annotation of human milk oligosaccharides in NMR spectra of human milk has been previously reported.^18,52,53^ A figure comparing NMR spectra of Se+ and Se- milk together with spectra of pure oligosaccharide standards is shown in Supplementary Figure S1. For the determination of the secretor phenotype, the identification of 2’FL was accomplished through identifying a combination of the methyl peaks from the fucose ring centered at 1.22 ppm combined with a set of peaks at 5.30 ppm. Information on peaks used to quantify other oligosaccharides is provided in the legend to Supplementary Figure S1. In this study, when [2’FL] ≥ 200 μM, milk secretor status was assigned as Se+, while milk samples with [2’FL] <200 μM were assigned as Se-.

### Fecal microbial DNA extraction and library preparation

The pellet of each fecal sample from above was used for DNA extraction according to the Human Microbiome Project (HMP) protocol with minor modifications using the MoBio PowerLyzer PowerSoil DNA isolation kit (MoBio, Carlsbad, CA).^38,54^ DNA purity was determined spectrophotometrically using a NanoDrop 2000C Spectrophotometer (Thermo Fisher Scientific, Waltham, MA, USA). One negative control sample using PCR-grade water (MoBio, Carlsbad, CA) was prepared for each batch.

The V4 hypervariable region of the 16S rRNA gene was targeted using a two-step PCR protocol. In step 1, the V4 region was amplified using F515/R806 primers modified to contain an Illumina overhang sequence and a 0–5 bp spacer on the 5’ end. The modified F515 forward primer sequence was as follows: 5’- *TCGTCGGCAGCGTCAGATGTGTATAAGAGACAG[spacer]GTGCCAGCMGCCGCGGTAA*-3’, and the modified R806 reverse primer sequence was as follows: 5’*-GTCTCGTGGGCTCGGAGATGTGTATAAGAGACAG[spacer]GGACTACHVGGGTWTCTAAT*-3’. Before PCR, the forward and reverse primers were diluted to 10 μM. The PCR reactions were performed in 15 µL reaction volumes containing 4 μL DNA template, 0.75 μL DMSO (Fisher Scientific, Waltham, MA), 3 μL 5X KAPA HiFi Buffer (KAPA Biosystems, Woburn, MA), 0.45 μL dNTP Mix (10 mM), 0.3 μL KAPA HiFi HotStart Polymerase (KAPA Biosystems, Woburn, MA), 5.7 μL PCR-grade water (MoBio, Carlsbad, CA), and primers (0.4 μL for each). The amplified DNA products in this step were diluted 1:10 using PCR water and mixed well. In step 2, an 8 bp index was used to multiplex the samples in both the forward and reverse primers. The forward indexing primer sequence was as follows: *AATGATACGGCGACCACCGAGATCTACACXXXXXXXXTCGTCGGCAGCGTC*, and the reverse indexing primer sequence was as follows: *CAAGCAGAAGACGGCATACGAGATXXXXXXXXGTCTCGTGGGCTCGG* (X indicates the positions of the 8-bp indices). The indexing primers were diluted to 5 μM before using. The PCR reactions were performed in duplicate in 20 µL reaction volumes containing 1 μL diluted DNA template from step 1, 1 μL DMSO (Fisher Scientific, Waltham, MA), 1 μL 5X KAPA HiFi Buffer (KAPA Biosystems, Woburn, MA), 0.6 μL dNTP Mix (10 mM), 0.4 μL KAPA HiFi HotStart Polymerase (KAPA Biosystems, Woburn, MA), 9 μL PCR-grade water (MoBio, Carlsbad, CA), and primers (2 μL for each). In both steps 1 and 2, the PCR reactions consisted of an initial denaturation at 95°C for 5 min followed by a 10-cycle program of 20 s at 98°C for denaturation, 15 s at 55°C for annealing, 60 s at 72°C for primer extension and a final extension of 72°C for 10 min.

Amplified PCR products from step 2 were quality checked by gel electrophoresis. The band intensity (around 430 bp) was visualized using SYBR safe DNA stain (Invitrogen), and its quantity (in ng/µL) was estimated using a molecular ladder with known concentration (BioRad EZ ladder 1 kb) through ImageLab software (v5.2.1, BioRad, Hercules, CA). Amplicons were pooled in equimolar ratios and purified using the QIAquick PCR Purification Kit (QIAGEN, Hilden, Germany) using a modified protocol from the manufacturer’s instruction. A purified amplicon library was quality checked by Bioanalyzer and submitted to the UC Davis Genome Center DNA Technologies Core for 300 bp paired-end sequencing on the Illumina MiSeq platform.

### Analysis of 16s amplicon sequence

Sequencing reads were demultiplexed by the UC Davis Genome center DNA Technologies Core after sequencing based on the index sequences provided upon library submission. Sequence reads without a corresponding barcode and primer sequence were discarded. Spacers and the V4 region primers were removed using the *cutadapt* function in *cutadapt* module (version 1.8.3). Reads were then split into forward (R1) and reverse (R2) reads before feeding into the *fastqc* and *multiqc* function to evaluate reading quality. DADA2 (version 1.12.1) was then used to filter, trim, merge reads and assign taxonomy. Briefly, forward and reverse reads were trimmed to 200 and 150 bases with a maxEE of 2, respectively. After error rate learning and sample inference, paired reads were merged, and sequences with a length between 250 and 258 bp were kept. Taxonomy was assigned using the SILVA 16S rRNA database version 138 formatted for DADA2.^55,56,57^ The ASV table and taxonomy table were then exported from the DADA2 pipeline for further analysis in R (version 4.0.3).

### Statistical analysis

Statistical analyses and graphical generation were performed using the R programming environment version 4.0.3. The ASV table generated from the DADA2 pipeline was used to calculate the relative abundance of each genus and α-diversity matrices using the *phyloseq* package. The generalized log transformation (defined as *log(y + 1))* was applied to all metabolomics data.

The infant length-for-age z-score (LAZ), weight-for-age z-score (WAZ), weight-for-length z-score (WLZ), and infant body-mass-index-for-age z-score (BMIZ) were calculated using the *anthro* package from the CRAN repository.

Principal coordinate analysis (PCoA) of microbiome data was computed using the *pcoa* package based on the Bray distance, while PCoA of metabolome data was done based on Euclidean distances. The centroids of each cluster (centroid of mass) were calculated as the average of PC1 and PC2 for each group. The ellipses were constructed based on a multivariate normal distribution at a 95% confidence level.

Differential analysis for the microbiome was computed at the genus level using analysis of composition of microbiomes (ANCOM), and infant age was set as a covariate. The false discovery rate (FDR) was controlled using the Benjamini–Hochberg procedure. For metabolomics data, the significance between secretor status was evaluated using the Mann–Whitney Test (*wilcox.test* function) and p-values from the Mann–Whitney test were then adjusted for FDR (*p.adjust(, method = fdr’)*). The overall level of significance was set at *p* < .05.

The *cliff.delta* function from the *effsize* package was used to evaluate the effect size between the Se+ and Se- group using Cliff’s delta (δ) statistics. The 95% confidence interval of each computed Cliff’s delta was further estimated. The threshold of negligible, small, and large δ were assessed according to Romano *et al*. ^58^ where |δ|<0.147 corresponds to negligible, 0.147<|δ|<0.33 corresponds to small, 0.33 <|δ|<0.474 corresponds to medium, and |δ|>0.474 large effect sizes.

The Spearman correlation coefficient (R) was computed using *cor*(*method = “spearman”*) to evaluate the strength of correlations. All plots were generated using *ggplot2*.

## Supplementary Material

Supplemental Material

Supplemental Material

## Data Availability

Metabolome data are available as supplementary tables. Squencing data are available at Dryad: https://datadryad.org/stash/share/tMOWGLIvAJo2yx4QrfCyRxexwL6ewSixWF-sNm74soo. ((doi:10.25338/B8V93G))

## Abbreviations

HMO: human milk oligosaccharides, FUT2: α-1,2-fucosyltransferase 2, FUT1: α-1,2-fucosyltransferase 1, LNFP I: lacto-N-fucopentose I, LNFP II: lacto-N-fucopentose II, LNFP III: lacto-N-fucopentose III, LDFT: lactodifucotetraose, 2’FL: 2’fucosyllactose, 3FL: 3-fucosyllactose, LNnT: lacto-N-neotetraose, GlcNAc: N-Acetylglucosamine, EBF: exclusively breastfed, ASVs: amplicon sequence variants, DTM: dose-to-mother, SCFAs: short chain fatty acids, Se+: secretor, Se-: non-secretor, PCoA: Principal coordinate analysis, FDR: false discovery rate, ANCOM: analysis of composition of microbiomes, BMI: body mass index, LAZ: length-for-age z-score, WAZ: weight-for-age z-score, WLZ: weight-for-length z-score, BMIZ: BMI-for-age index, WEIRD: western, educated, industrialized, rich, and democratic
