# Supplementary material for: Impact of milk secretor status on the fecal metabolome and microbiota of breastfed infants
Source: Gut Microbes. 2023 Sep 23;15(2):2257273. doi: 10.1080/19490976.2023.2257273 (PMC10519369; doi:10.1080/19490976.2023.2257273)
Supplement: Supplemental Material [file KGMI_A_2257273_SM8374.docx]

Impact of milk secretor status on the fecal metabolome and microbiota of breastfed infants

Aidong Wang, Aly Diana, Sofa Rahmannia, Rosalind S Gibson, Lisa A Houghton, and Carolyn M Slupsky*

**Supplementary Figures**

**Supplementary Figure 1**. Comparison of NMR spectra from milk assigned as either secretor milk (Se+) or non-secretor milk (Se-) with spectra of pure oligosaccharides. (A) The sugar region downfield of water (5.0 to 5.5 ppm). (B) The methyl region (1.1 to 1.4 ppm). Resonances from 2’- fucosyllactose (2’-FL), 3-fucosyllactose (3FL), LDFT (lactodifucotetraose), lacto-N-fucopentaose I (LNFP I), LNFP II, and LNFP III are shown as standards. Identification and quantification of metabolites were made as follows: For 2’-FL, LDFT and 3FL, a combination of the methyl peaks and the peaks centered between 5.25 and 5.45 were used; for LNFP I, the doublet at 5.18 ppm in combination with the methyl group at 1.22 ppm, and peaks centered at 2.05 and 4.13 ppm were used (peaks at 2.05 and 4.13 are not shown in figure); for LNFP II the doublet just above 5.0 ppm was used, and the methyl peaks were fit below 3FL; for LNFP III, a combination of the methyl peaks and the doublet just above 5.1 ppm was used. For LNFP I, II and III, amide hydrogen peaks between 8.34 and 8.44 were also used for identification (not shown).

**Supplementary Figure 2**. Comparison of relative abundance of genera represented at a level of over 1% in the feces of infants at different ages consuming secretor (cyan) or non-secretor (red) milk. (A) Infants younger than 3.75 months (n=28 in each group; 12 female, and 16 male in the Se- group, 15 female and 13 male in the Se+group) and (B) Infants at 3.75 months or older (n=19 in each group; 10 female, and 9 male in Se- group, and 11 female, and 8 male in Se+ group). No genera showed significant difference between infants consuming secretor (cyan) or non-secretor (red) milk via ANCOM analysis.

**Supplementary Figure 3**. Forest plot illustrating the Cliff’s delta effect size analysis of fecal metabolites comparing infants consuming secretor (cyan) or non-secretor (red) milk. (A) Infants younger than 3.75 months (n=56, with 28 in each group; 12 female, and 16 male in the Se- group, 15 female and 13 male in the Se+group) and (B) Infants at 3.75 months or older (n=38, with 19 in each group; 10 female, and 9 male in the Se- group, 11 female, and 8 male in the Se+ group). All metabolites had pFDR > 0.05 assessed with a Mann-Whitney U test.

**Supplementary Figure 4**. Spearman correlation between fecal metabolites and the relative abundance of Bifidobacterium and Streptococcus of 50 randomly selected infants consuming milk from secretor mothers.


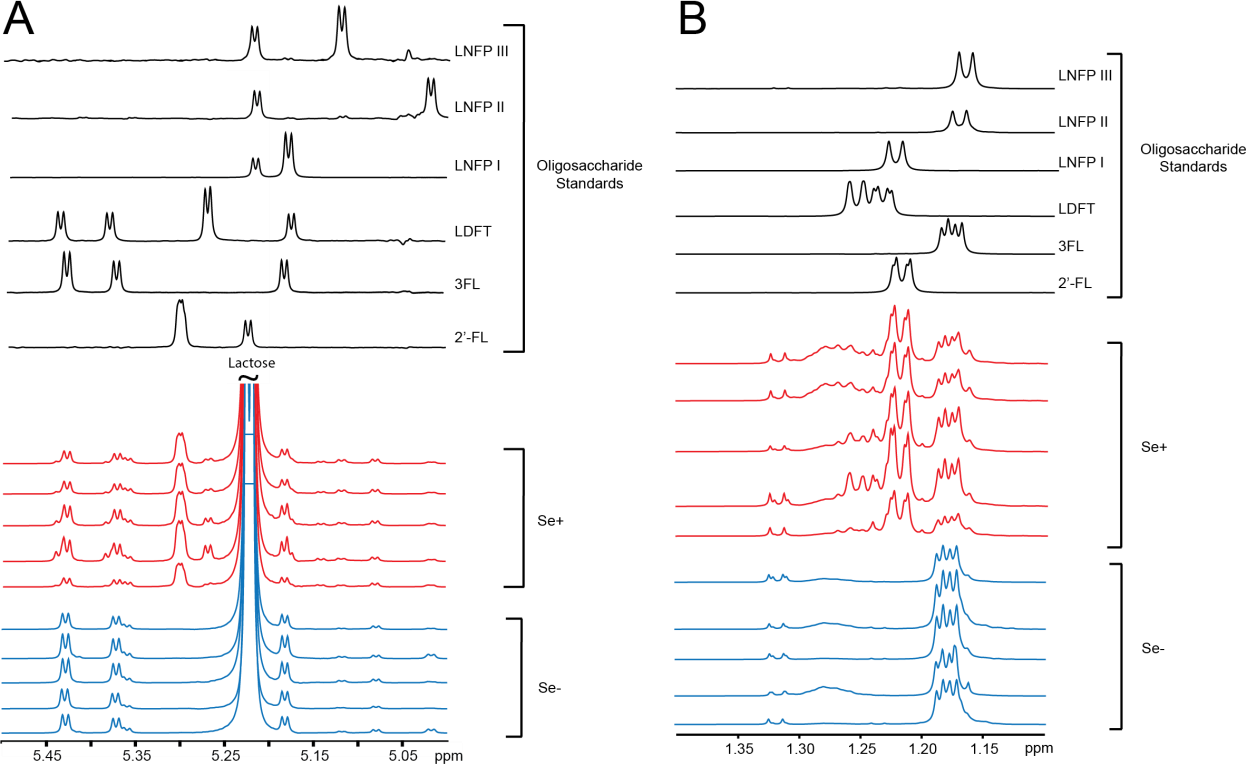


**Supplementary Figure 1**. Comparison of NMR spectra from milk assigned as either secretor milk (Se+) or non-secretor milk (Se-) with spectra of pure oligosaccharides. (A) The sugar region downfield of water (5.0 to 5.5 ppm). (B) The methyl region (1.1 to 1.4 ppm). Resonances from 2’- fucosyllactose (2’-FL), 3-fucosyllactose (3FL), LDFT (lactodifucotetraose), lacto-N-fucopentaose I (LNFP I), LNFP II, and LNFP III are shown as standards. Identification and quantification of metabolites were made as follows: For 2’-FL, LDFT and 3FL, a combination of the methyl peaks and the peaks centered between 5.25 and 5.45 were used; for LNFP I, the doublet at 5.18 ppm in combination with the methyl group at 1.22 ppm, and peaks centered at 2.05 and 4.13 ppm were used (peaks at 2.05 and 4.13 are not shown in figure); for LNFP II the doublet just above 5.0 ppm was used, and the methyl peaks were fit below 3FL; for LNFP III, a combination of the methyl peaks and the doublet just above 5.1 ppm was used. For LNFP I, II and III, amide hydrogen peaks between 8.34 and 8.44 were also used for identification (not shown).


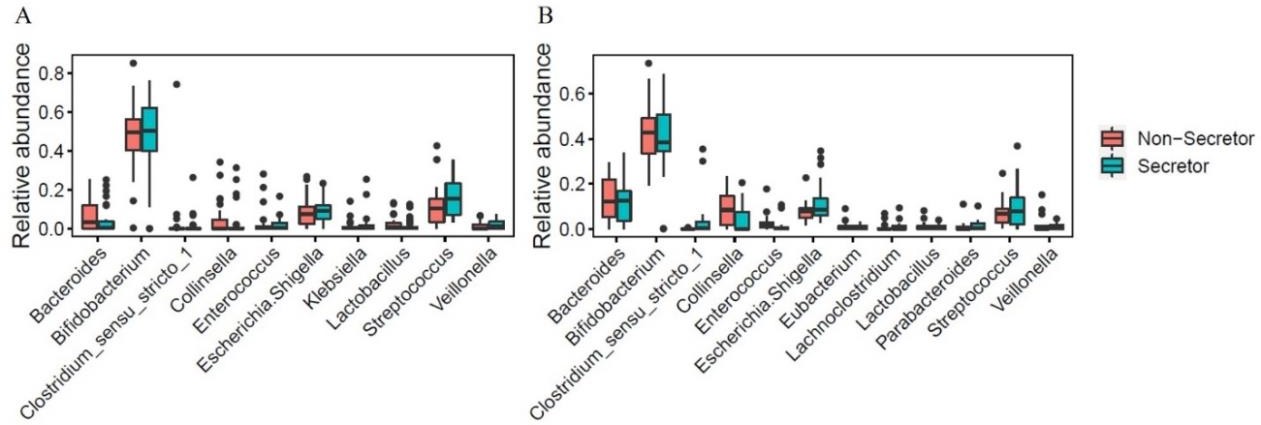


**Supplementary Figure 2**. Comparison of relative abundance of genera represented at a level of over 1% in the feces of infants at different ages consuming secretor (cyan) or non-secretor (red) milk. (A) Infants younger than 3.75 months (n=28 in each group; 12 female, and 16 male in the Se- group, 15 female and 13 male in the Se+group) and (B) Infants at 3.75 months or older (n=19 in each group; 10 female, and 9 male in Se- group, and 11 female, and 8 male in Se+ group). No genera showed significant difference between infants consuming secretor (cyan) or non-secretor (red) milk via ANCOM analysis.


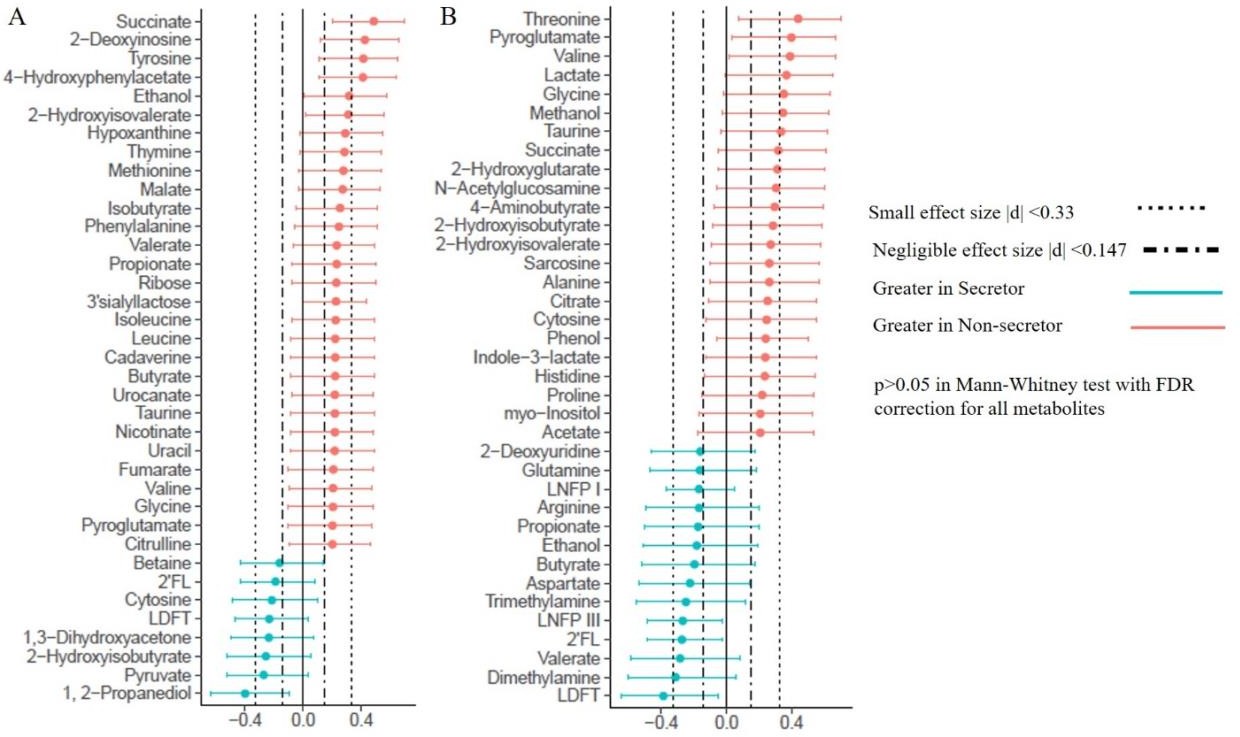


**Supplementary Figure 3**. Forest plot illustrating the Cliff’s delta effect size analysis of fecal metabolites comparing infants consuming secretor (cyan) or non-secretor (red) milk. (A) Infants younger than 3.75 months (n=56, with 28 in each group; 12 female, and 16 male in the Se- group, 15 female and 13 male in the Se+group) and (B) Infants at 3.75 months or older (n=38, with 19 in each group; 10 female, and 9 male in the Se- group, 11 female, and 8 male in the Se+ group). All metabolites had pFDR > 0.05 assessed with a Mann-Whitney U test.


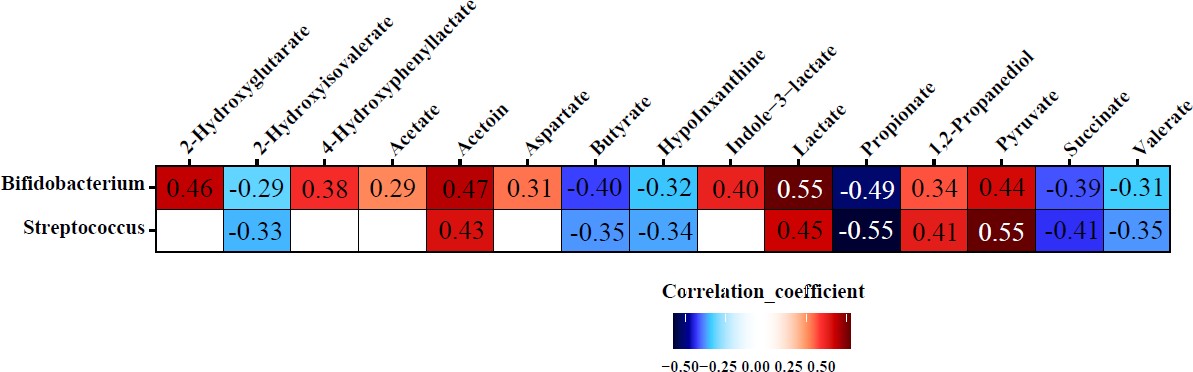


**Supplementary Figure 4**. Spearman correlation between fecal metabolites and the relative abundance of *Bifidobacterium* and *Streptococcus* of 50 randomly selected infants consuming milk from secretor mothers.
